# Versican and vascular endothelial growth factor expression levels in peritoneal metastases from colorectal cancer are associated with survival after cytoreductive surgery and hyperthermic intraperitoneal chemotherapy

**DOI:** 10.1007/s10585-016-9779-9

**Published:** 2016-02-12

**Authors:** N. R. Sluiter, E. M. V. de Cuba, R. Kwakman, W. J. H. J. Meijerink, P. M. Delis-van Diemen, V. M. H. Coupé, J. A. M. Beliën, G. A. Meijer, I. H. J. T. de Hingh, E. A. te Velde

**Affiliations:** Section of Surgical Oncology and Digestive Surgery, Department of General Surgery, VU University Medical Centre, De Boelelaan 1117, 1081 HV Amsterdam, The Netherlands; Department of Pathology, VU University Medical Centre, De Boelelaan 1117, 1081 HV Amsterdam, The Netherlands; Department of Epidemiology and Biostatistics, VU University Medical Centre, De Boelelaan 1117, 1081 HV Amsterdam, The Netherlands; Department of Pathology, NKI-Antoni van Leeuwenhoek, Plesmanlaan 121, 1066 CX Amsterdam, The Netherlands; Department of Surgery, Catharina Hospital Eindhoven, Michelangelolaan 2, 5623 EJ Eindhoven, The Netherlands

**Keywords:** Peritoneal metastases, Colorectal cancer, HIPEC, Versican, VEGF, Angiogenesis, Biomarkers

## Abstract

Cytoreductive surgery (CRS) and hyperthermic intraperitoneal chemotherapy (HIPEC) can increase survival of colorectal cancer (CRC) patients with peritoneal metastases (PM). This treatment is associated with high morbidity and mortality rates. Therefore, improvement of patient selection is necessary. Assuming that the clinical phenotype is dictated by biological mechanisms, biomarkers could play a crucial role in this process. Since it is unknown whether and to what extent angiogenesis influences the course of disease in patients with PM, we investigated the expression of two angiogenesis-related markers and their relation to overall survival (OS) in CRC patients after CRS and HIPEC. Clinicopathological data and tissue samples were collected from 65 CRC patients with isolated metastases to the peritoneum that underwent CRS and HIPEC. Whole tissue specimens from PM were evaluated for versican (VCAN) expression, VEGF expression and microvessel density (MVD) by immunohistochemistry. The relation between these markers and OS was assessed using univariate and multivariate analysis. Associations between VEGF expression, VCAN expression, MVD and clinicopathological data were tested. High stromal VCAN expression was associated with high MVD (p = 0.001), better resection outcome (p = 0.003) and high T-stage (p = 0.027). High epithelial VCAN expression was associated with MVD (p = 0.007) and a more complete resection (p < 0.001). In multivariate analysis, simplified peritoneal cancer index (p = 0.001), VEGF expression levels (p = 0.012), age (p = 0.030), epithelial VCAN expression levels (p = 0.042) and lymph node status (p = 0.053) were associated with OS. Concluding, VCAN and VEGF were associated with survival in CRC patients with PM after CRS and HIPEC. Independent validation in a well-defined patient cohort is required to confirm the putative prognostic role of these candidate biomarkers.

**Electronic supplementary material:**

The online version of this article (doi:10.1007/s10585-016-9779-9) contains supplementary material, which is available to authorized users.

## Introduction

Cytoreductive surgery (CRS) combined with hyperthermic intraperitoneal chemotherapy (HIPEC) is the preferred therapeutic strategy for patients with isolated peritoneal metastases (PM) originating from colorectal cancer (CRC) [1, 2]. This approach results in a median survival ranging from 33 to 45 months [3–7]. A recent meta-analysis even suggests that the combination of CRS and HIPEC compared to treatment with modern systemic chemotherapy potentially improves outcome in a carefully selected group of patients with both PM and liver metastases [8].

Treatment with CRS and HIPEC has considerable morbidity and mortality rates of 15–34 and 5 % respectively [3, 6, 9, 10], with anastomotic leakage being the most common cause of mortality. Consequently, a relatively long median hospital stay of 16 days is observed [5]. The high morbidity and mortality rates significantly impact the quality of life [7] and thus selection of patients that will benefit most from this treatment is key. Prediction of patient response to the surgical procedure and chemotherapeutic compounds will help bring the proper treatment to the right patient, providing a much needed step forward towards personalised medicine [11]. Assuming that the clinical phenotype of this disease is ultimately dictated by biological mechanisms, read-outs of these mechanisms (i.e., biomarkers) might aid in predicting treatment outcomes [12].

More specifically, identifying biomarkers requires knowledge of molecules that contribute to peritoneal dissemination. Peritoneal dissemination is viewed as a multistep process, in which the establishment of a metastatic lesion ultimately depends on colonisation of cancer cells and the creation of a metastatic niche [11]. At this step, the formation of a tumour-specific microenvironment is pivotal. Interactions between cancer cells and their surrounding stroma provide growth and survival signals necessary to evade apoptosis, to enable invasion and migration, and to provide oxygen and nutrients through angiogenesis [13–16]. Angiogenesis is crucial for tumour growth beyond 150–200 μm from the nearest blood vessel and for progression of micrometastases to macrometastases [14, 17, 18]. Consequently, angiogenesis influences tumour progression and is associated with higher morbidity and mortality rates in HIPEC patients, as is suggested by several authors [19, 20]. Angiogenesis depends on complex interactions between several cell types and extracellular matrix (ECM) components [17]. The exact interactions required for this process are not fully understood. In an other study we did not find correlations between the angiogenesis related markers hypoxia-inducible factor 1α (HIF1α), stromal derived factor 1 (SDF1), CXC chemokine receptor 4 (CXCR4) and vascular endothelial growth factor (VEGF) [19]. Several other studies, however, suggest a role for versican (VCAN) [21–24]. VCAN is a sulphate proteoglycan overexpressed in a variety of human malignant tumours, including CRC [21, 25–31]. VCAN-mediated angiogenesis possibly depends upon interaction with VEGF. VEGF-A induced microvessel formation was accompanied by VCAN degradation and subsequent resynthesis [32]. In human astrocytoma cells VCAN was shown to form a complex with fibronectin and VEGF. This complex enhanced endothelial cell adhesion, proliferation and migration, possibly providing the molecular basis by which VCAN promotes angiogenesis [33]. It is not known, however, whether and to what extent VCAN influences the course of disease in PM patients. Additionally, the relation between VCAN expression, VEGF expression and angiogenesis has not yet been investigated in PM. The aim of the present study is to assess the possible role of VCAN as a prognostic biomarker in CRC patients with PM who underwent treatment with CRS and HIPEC. Secondly, correlations between VCAN and VEGF expression levels, microvessel density (MVD) and clinicopathological parameters were evaluated.

## Methods

### Treatment: CRS and HIPEC

In two tertiary referral centres, the Catharina Hospital Eindhoven from 2007 to 2010 and the VU University Medical Centre Amsterdam from 2010 to June 2013, 78 patients with PM of CRC were treated with CRS and HIPEC with curative intent. Both institutions performed the CRS and HIPEC procedure by following the same standardised protocol [5]. Complete debulking, stripping of the affected parietal peritoneum, and removal of the omentum and adnexa was performed as described by Sugarbaker et al. [1]. When deemed necessary, multi-organ resections were carried out. Subsequently, intra-abdominal lavage with Mitomycin C (MMC), 35 mg/m^2^ body surface, was performed with a target intraperitoneal temperature of 39–41 °C for 90 min.

### Patient selection and data collection

Patients with PM of non-colorectal origin (n = 2) were excluded. Specimens of 11 patients were not eligible for analysis due to loss of tissue or technical difficulties, leaving 65 patients eligible for further analysis.

Clinicopathological characteristics and follow-up data were collected from patient records of both institutions (Table 1). Resection outcome was determined according to the maximal size of residual tumour tissue. An R1 resection was recorded when no macroscopically visible tumour was left behind, an R2a resection when the residual macroscopic tumour was smaller than 2.5 mm and an R2b resection when macroscopically tumour deposits larger than 2.5 mm remained [34].

**Table 1:** Clinicopathological characteristics of 65 colorectal cancer patients with peritoneal metastases

Characteristic	n/mean/median	%/SD/range
General characteristics		
All	65	
Mean OS (months, range)	34.4	29.4–39.2
Gender		
Male (%)	25	38.5
Female (%)	40	61.5
Age at CRS and HIPEC (years)		
Mean (SD)	59.6	11.6
Median (range)	62	31–78
Follow-up after CRS and HIPEC (months)		
Mean (SD)	21.0	10.8
Median (range)	21.0	2–50
Primary tumour characteristics		
Location primary tumour		
Colon (%)	49	75.4
Rectum (%)	5	7.7
Rectosigmoid (%)	11	16.9
Tumour differentiation		
Poor (%)	11	17.0
Moderate (%)	22	33.8
Well (%)	3	4.6
Unknown (%)	29	44.6
Tumour histology		
Adenocarcinoma (%)	39	60.0
Mucinous (%)	22	33.8
Signet cell (%)	4	6.2
Lymph node involvement		
Yes (%)	46	70.8
No (%)	17	26.2
Unknown (%)	2	3.1
T-stage primary tumour		
T1 (%)	1	1.5
T2 (%)	2	3.1
T3 (%)	27	41.5
T4 (%)	34	52.3
Unknown (%)	1	1.5
Stage primary tumour		
Stage 1 (%)	1	1.5
Stage 2 (%)	10	15.4
Stage 3 (%)	18	27.7
Stage 4 (%)	35	53.8
Unknown (%)	1	1.5
PM characteristics		
Synchronous PM		
Yes (%)	35	53.8
No (%)	30	46.2
Simplified PCI		
<2 (%)	1	1.5
2–4 (%)	42	64.6
5 (%)	14	21.5
>5 (%)	8	12.3
Resection outcome		
R1 (%)	37	56.9
R2a (%)	22	33.8
R2b (%)	6	9.2
Adjuvant chemotherapy		
Yes (%)	41	63.1
No (%)	24	36.9
Pre-operative chemotherapy 6 months before HIPEC		
Yes (%)	17	26.2
No (%)	48	73.8

*OS* overall survival, *CRS* cytoreductive surgery, *HIPEC* hyperthermic intraperitoneal chemotherapy, *PM* peritoneal metastases, simplified *PCI* peritoneal cancer index

Tumour specimens were collected during CRS and preserved as formalin-fixed paraffin-embedded (FFPE) tissue. Collection, storage and use of clinicopathological data and tissue specimens were performed in compliance with the ‘Code for Proper Secondary Use of Human Tissue’ in The Netherlands.

### Assays and immunohistochemistry protocols

Sections of histologically confirmed PM from CRC (4 μm) were mounted on glass slides and immunohistochemically stained for VCAN, VEGF and CD31. After deparaffinisation and rehydration, endogenous peroxidases were blocked with 0.3 % hydrogen peroxide in methanol. Tissue sections were treated with 10 mM citrate buffer (pH 6.0) in a microwave for 30 min at 90 Watts for the VCAN staining and for 5 min at maximum power followed by 10 min at 360 Watts for the VEGF and CD31 staining. Sections were subsequently incubated overnight at 4 degrees Celsius with primary mouse anti-VCAN antibody (clone 2-B-1, Seikagaku, Tokyo, Japan; dilution 1:300) and anti-VEGF antibody labelling the VEGF-121, VEGF-165 and the VEGF-189 isoforms (clone v91, Dako, Carpinteria, USA, dilution 1:50) and incubated for 1 h at room temperature with anti-CD31 antibody (clone JC70A, Dako, Carpinteria, USA, dilution 1:50). Signals were visualised with a horseradish peroxidase-coupled anti-mouse polymer (Envision, Dako, Heverlee, Belgium) for VCAN and a poly-HRP-GAM/R/R IgG (PowerVision) for VEGF and CD31. This procedure was followed by diaminobenzidine (Dako) and Mayer’s haematoxylin counterstain.

### Evaluation of protein expression

**Fig. 1:**
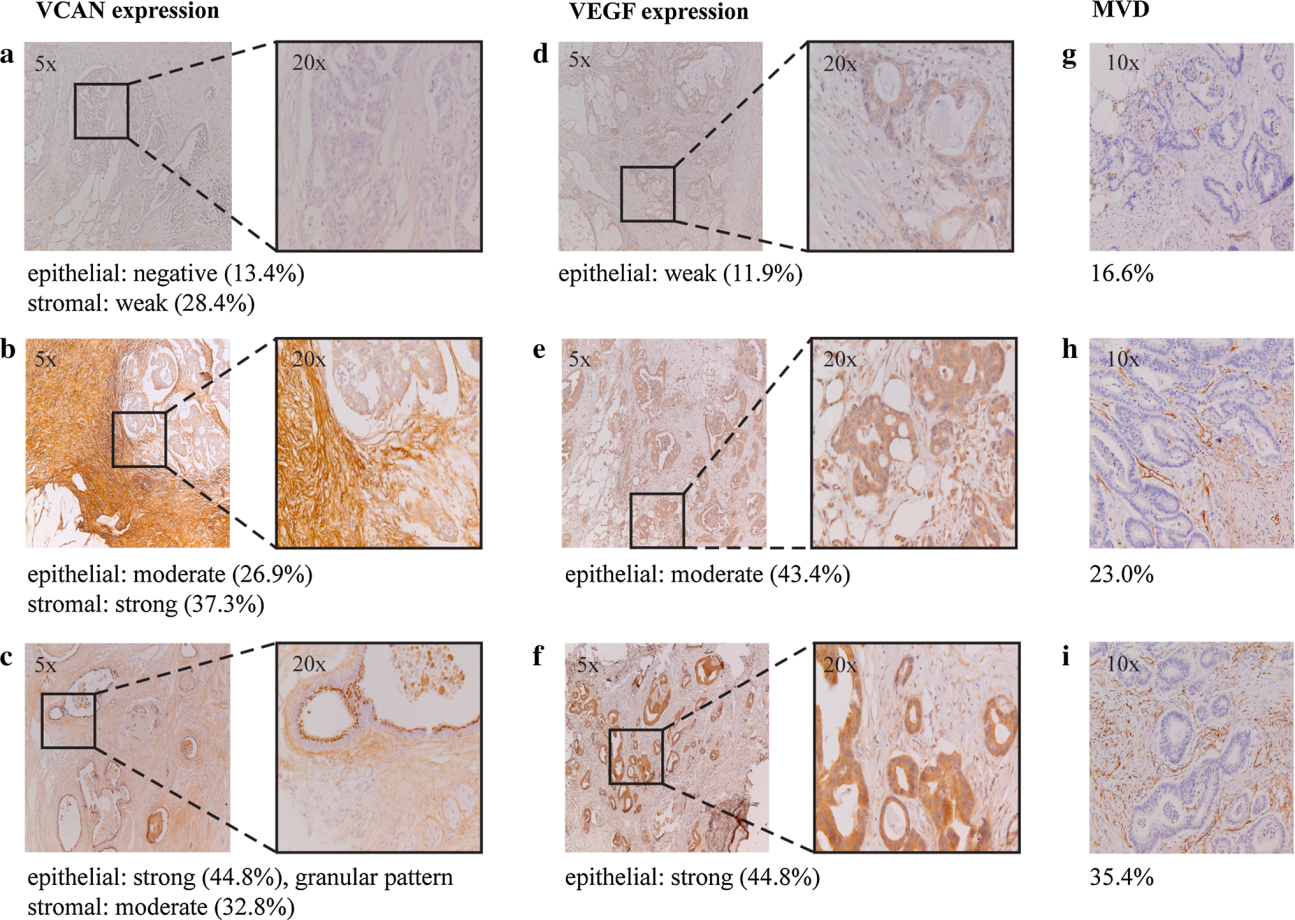
Representative examples of versican (VCAN) (**a**, **b**, **c**) and vascular endothelial growth factor (VEGF) (**d**, **e**, **f**) expression patterns and microvessel density (MVD) (**g**, **h**, **i**) in peritoneal lesions. The magnification is shown on the micrographs. Intensities were classified as negative, weak, moderate or strong. Scoring percentages of all markers are listed next. Stromal VCAN expression: negative (1.5 %), weak (28.4 %), moderate (32.8 %) and strong (37.3 %). Epithelial VCAN expression: negative (13.4 %), weak (14.9 %), moderate (26.9 %) and strong (44.8 %). VEGF expression: weak (11.9 %), moderate (43.3 %) and strong (44.8 %)

Initially, tissue samples were scored for VCAN and VEGF intensity in four categories (negative, weak, moderate and strong, Fig. 1) but dichotomised into two categories: negative and weak as low expression versus moderate and strong as high expression for VCAN and weak and moderate as low expression versus strong as high expression for VEGF. Cut-offs were determined that discriminated best between the low and high expressing groups using the Kaplan–Meier method. Scoring was done using a ×20 magnification (×20/0.45, diameter 0.98 mm), depending on lesion size. Staining pattern and the location of protein expression were assessed. VCAN staining was expressed in the cytoplasm of tumour cells and in the stroma surrounding the tumour cells. Hence, both stromal and epithelial VCAN were analysed. Since VEGF was predominantly seen in the cytoplasm of tumour cells, its intensity was scored in the cytoplasmic compartment. All tissue sections were scored and examined blinded to clinicopathological data. Fifteen per cent of sections were analysed independently in a blinded fashion by a second investigator (NRS and EMVdC) with high inter-observer agreement for all stainings (Cohen’s weighted kappa value, VEGF K_w_ = 0.82, epithelial K_w_ = 0.65, stromal VCAN K_w_ = 0.74).

MVD was determined by automated image analysis of CD31 staining patterns. In short, complete slides were scanned with a digital Mirax slide Scanner system (3DHISTECH, Budapest, Hungary). The scan resolution of all pictures at ×20 was 0.23 μm. Next, areas with tumour tissue were manually selected using the Panoramic Viewer software (3DHISTECH) and exported in the TIFF image-format. A computerised morphometric analysis of the CD31 stained slides was executed using ImageJ. The CD31 staining was used to quantify the amount of microvessels per area as the percentage of the field of view that was CD31 positive [35]. Morphometric measurements were performed without knowledge of sample identity. For survival analysis, MVD was dichotomised as ‘low’ and ‘high’ if respectively less than 31.0 % -the mean MVD percentage- and more than 31.0 % of the analysed area was stained positive for CD31.

### Statistical analysis

Associations between the staining intensities of the markers and clinicopathological variables were studied by means of the Spearman’s correlation coefficient (two continuous variables), the Fisher’s Exact Test (two categorical/dichotomous variables) or the Kruskal–Wallis test (a continuous non-normally distributed variable with a categorical/dichotomous variable) [36]. Associations were considered statistically significant for p values smaller than or equal to 0.05 (two sided test).

Overall survival (OS) was defined as time in months from date of surgery to death from any cause. Patients were censored at date last known to be alive. Univariate associations between OS and potential prognostic variables were studied by means of Kaplan–Meier analysis and tested using the log-rank test (Table 3). Variables with a p value ≤0.1 and possible relevant markers were included in a multivariate Cox regression analysis. Variable selection in the Cox model was done using backward selection with a threshold p value of 0.1 for exclusion from the model.

All data were reported according to the REMARK guidelines. Statistical analyses were performed using the package for the social sciences version 20 for OsX (IBM, Armonk, NY, USA).

## Results

### Associations between VCAN, VEGF, MVD and clinicopathological characteristics

Significant associations between markers and clinicopathological variables are listed in Table 2. A complete overview of all associations is shown in supplementary Table 1a–d. VCAN was expressed in both the epithelial and the stromal compartment. Stromal VCAN was negative in 1.5 %, weak in 28.4 %, moderate in 32.8 % and strong in 37.3 % of patients (Fig. 1a–c). The group patients with high stromal VCAN expression had an MVD of 34.4 % (SD 12.6) and the group with low stromal VCAN expression an MVD of 22.4 % (SD 7.7) (p = 0.001). Furthermore, we found high VCAN expression in the stromal compartment to be associated with better resection outcome (p = 0.003, odds ratio [OR] 0.17, 95 % CI 0.053–0.53, Table 2) and higher T-stage of the primary tumour (p = 0.027, OR 3.59, 95 % CI 2.40–5.37, Table 2).

**Table 2:** Significant associations between VCAN, VEGF, MVD and clinicopathological variables

		Low^ (%)	High^ (%)	p value	Odds ratio (95 % CI)
Stromal VCAN expression					
Resection outcome	R1	6 (28.6)	31 (70.5)	0.003*	0.168 (0.053–0.528)
	R2a/R2b	15 (71.4)	13 (29.5)		
T-stage	T1/T2	3 (15.0)	0 (0)	0.027*	3.588 (2.396–5.373)
	T3/T4	17 (85.0)	44 (100)		
MVD, % (SD)	–	22.4 (7.7)	34.4 (12.6)	0.001**	–
Epithelial VCAN expression					
Resection outcome	R1	4 (21.1)	33 (71.7)	<0.001*	0.105 (0.029–0.376)
	R2a/R2b	15 (78.9)	13 (46.6)		
MVD, % (SD)	–	22.9 (9.6)	33.9 (12.4)	0.007**	–

		Negative (%)	Not granular (%)	Granular (%)	p value	Odds ratio (95 % CI)
VCAN staining pattern						
Resection outcome	R1	3 (30.0)	7 (35.0)	27 (77.1)	0.001*	–
	R2a/R2b	7 (70.0)	13 (65.0)	8 (22.9)		
Gender	Male	4 (40.0)	3 (15.0)	18 (51.4)	0.028*	–
	Female	6 (60.0)	17 (85.0)	17 (48.6)		

* Fisher’s exact test; ** Kruskal–Wallis test

*^Low* negative and weak expression, *high* moderate and weak expression

Epithelial VCAN was negative in 13.4 %, weak in 14.9 %, moderate in 26.9 % and strong in 44.8 % (Fig. 1a–c). In the VCAN positive cells a cytoplasmic staining was seen, sometimes with a highly intense granular pattern, probably depicting the Golgi system [37]. In the patients with high epithelial VCAN expression an MVD of 33.9 % (SD 12.4) was seen and in the patients with low epithelial VCAN expression an MVD of 22.9 % (SD 9.6) (p = 0.007). Both epithelial VCAN (p < 0.001, OR 0.11, 95 % CI 0.029–0.38, Table 2) and a granular epithelial VCAN expression pattern (p = 0.001, Table 2) correlated with a more favourable resection outcome. In male patients, epithelial VCAN expression was mostly granular (p = 0.028, Table 2), which may be related to an androgen-mediated regulation of the VCAN gene [38].

VEGF expression in the cytoplasmic compartment of the tumour cells was detected in 100 % of the patients. In 11.9 % of these patients, weak VEGF expression, in 43.3 % moderate VEGF expression and in 44.8 % strong VEGF expression (Fig. 1d–e) was observed. No association was observed between epithelial VEGF expression and MVD (p = 0.596), epithelial VCAN expression (p = 0.791) or stromal VCAN expression (p = 0.595).

The mean and median MVD were 31.0 % (SD 12.4 %) and 28.9 % (range 7.5–61.0 %) respectively. MVD correlated to stromal and epithelial VCAN, but did not show associations with other markers or clinicopathological characteristics.

### VEGF and epithelial VCAN are associated with OS in CRC patients with PM

**Fig. 2:**
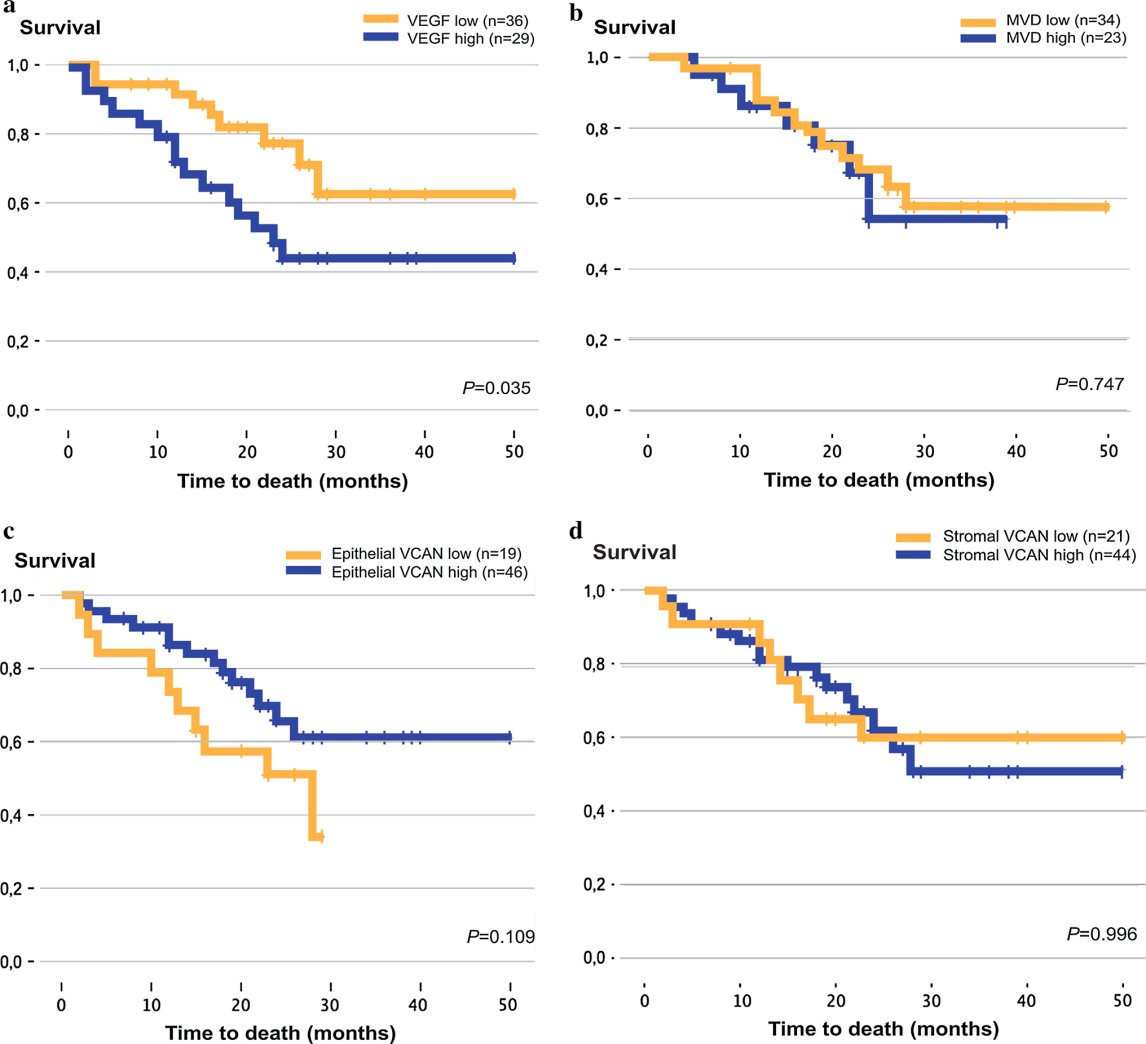
Kaplan–Meier curves of all patients. The *graphs* depict the curves according to the dichotomised vascular endothelial growth factor (VEGF) (**a**), microvessel density (MVD) (**b**), epithelial versican (VCAN) (**c**) and stromal VCAN (**d**) categories

Mean OS in the patient cohort was 34.4 months (95 % CI 29.4–39.2, Table 1). Mean survival in the group patients with low VEGF expression was 38.3 months and in the group patients with high VEGF expression 29.2 months (p = 0.035). Mean survival in patients with high epithelial VCAN expression was 36.8 months and in patients with low epithelial VCAN expression 20.1 months (p = 0.109). Furthermore, in patients with low and high stromal VCAN expression a mean survival of 34.9 and 33.8 months was observed respectively (p = 0.996, Fig. 2d). Mean survival in patients with low MVD was 36.5 months and in patients with high MVD mean survival was 29.0 months (p = 0.747, Fig. 2b).

Possibly important characteristics included in the multivariate analysis after being individually tested for correlation with OS were age (p = 0.056), lymph node status (p = 0.042), simplified peritoneal cancer index (PCI) [39] (p = 0.052), resection outcome (p = 0.021), preoperative chemotherapy 6 months before HIPEC (p = 0.007), VEGF (p = 0.035) and epithelial VCAN (p = 0.109) (Table 3).

**Table 3:** Final model resulting from multivariate survival analysis

Variable*	Hazard ratio	95 % CI	p value
Simplified PCI	0.235	0.097–0.570	0.001
VEGF expression	0.315	0.128–0.773	0.012
Age	1.048	1.004–1.094	0.030
VCAN expression	2.584	1.034–6.452	0.042
Lymph node status	0.296	0.086–1.014	0.053

* Variables tested for individual associations with OS (log rank): gender (p = 0.292), age (p = 0.056), histology class (p = 0.123), differentiation grade (p = 0.888), T-stage (p = 0.581), lymph node status (p = 0.042), stage (p = 0.270), location primary tumour (p = 0.807), synchronous metastases versus metachronous metastases (p = 0.132), PCI index (p = 0.052), resection outcome (p = 0.021), chemotherapy in the 6 month before CRS and HIPEC (p = 0.007), adjuvant chemotherapy (p = 0.724), MVD (p = 0.747), VEGF expression (p = 0.035), stromal VCAN expression (p = 0.996), epithelial VCAN expression (p = 0.109), a granular VCAN staining pattern (p = 0.779), simultaneous high VEGF and epithelial VCAN expression (p = 0.129) and simultaneous high VEGF and stromal VCAN expression (p = 0.158)

Variables that emerged as independent prognostic factors in the final model are listed in Table 3. In this model, high VEGF expression was significantly associated with worsened OS (p = 0.012, Table 3; Fig. 2a). In contrast, high epithelial VCAN expression was significantly associated with longer OS (0 = 0.042, Table 3; Fig. 2c). The observation of epithelial VCAN expression being significant in multivariate, but not in univariate analysis, is most probably attributable to the effect of confounding in the univariate analysis. This confounding effect is corrected for in multivariate analysis by including all possibly significant variables.

## Discussion

Although CRS and HIPEC increase survival rates of CRC patients with isolated PM [5–8], this treatment has considerable morbidity and mortality rates [3, 6, 9, 10]. Selection of patients that will benefit most from this treatment is key. Biomarkers could help in patient selection, preventing unnecessary morbidity in patients that will not benefit. Because it is not known whether and to what extent angiogenesis influences the course of disease in PM patients, the present study evaluated the expression of potential markers of angiogenesis—VCAN, VEGF and MVD—in these patients. In the multivariate analysis, VEGF and epithelial VCAN expression levels were found to be significantly associated with OS in CRC patients with PM after CRS and HIPEC (p = 0.012 and p = 0.042 respectively, Table 3; Fig. 2a, c). Furthermore, both high stromal and epithelial VCAN were related to increased microvessel density (MVD) (p = 0.001 and p = 0.007 respectively).

The currently observed association between VEGF and OS in CRC patients with PM is in accordance with several other clinical and preclinical studies demonstrating VEGF to have a role in PM formation and prognosis [20, 40–50]. The possible role of VEGF as a predictor of worse outcome corroborates the rationale for (neo-) adjuvant anti-VEGF based therapy in selected patients [20, 51, 52]. Neo-adjuvant administration of this drug was shown to improve outcome in patients after CRS and HIPEC [53]. Although a recent study described a twofold increase in morbidity in these patients after administration of bevacizumab [54], a meta-analysis including more than 3000 patients with metastasised CRC reported the amount and severity of treatment with bevacizumab to be acceptable [3].

The sulphate proteoglycan VCAN is thought to promote tumour development [21, 55] by mediating several processes such as proliferation [56–58], drug resistance [27, 56–62], cell adhesion [57–60], invasion [57, 58, 63] and angiogenesis [23, 24, 32, 33, 64, 65]. Angiogenesis in astrocytoma [33] and breast cancer [64] was suggested to result from interaction between VCAN and VEGF. In the present study, VEGF was not correlated to MVD, a finding supported by others [20]. Interestingly, stromal and epithelial VCAN levels did correlate with MVD. MVD is a measure of the number of vessels per high power field. Considering this number of vessels is not only determined by angiogenic factors, such as VEGF, but also by non-angiogenic factors such as oxygen and nutrient consumption rates, one might state that MVD does not necessarily adequately reflect the rate of angiogenesis [66]. Hence, it is possible that MVD is a marker of a more or less established vessel network, whilst VEGF is produced in the presence of hypoxic stimuli to stimulate angiogenesis [67]. Once vessels are established and an oxygen-rich environment is created, VEGF is no longer necessary for the formation of new vessels. In the presence of an established vessel network, other molecules, including VCAN, might be required for maintenance -and not creation- of a favourable microenvironment [14, 21]. To confirm this hypothesis, further experiments should focus on the exact mechanisms by which VEGF and VCAN possibly promote tumour growth in PM of CRC.

The present study revealed that epithelial VCAN is indicative for good survival in CRC patients with PM in multivariate analysis (p = 0.042, Table 3). This finding is supported by studies in primary CRC [37] and serous ovarian cancer [68]. The mechanism by which epithelial VCAN positively influences patient outcome might be related to tumour cell responses to chemotherapy [68]. Another study found cells transfected with V1 VCAN to be selectively sensitised to several apoptotic stimuli, for example to the chemotherapeutics etoposide and cisplatin [61]. The influence of molecules such as VCAN in tumour response to chemotherapy stresses the importance of systematically analysing interactions between cancer cells and their environment in order to identify mechanisms contributing to drug sensitivity. In peritoneal cancer patients, sensitivity of tumour cells to heated MMC might -at least in part- account for good outcome in patients with high epithelial VCAN expression. A recent paper found low Bloom syndrome protein (BLM) expression to be associated with high MMC sensitivity in CRC cell lines. Low BLM expression was thereby related to improved survival in CRC patients with PM [69]. Since identification of sensitivity and resistance mechanisms could lead to mechanism-based therapies and further stratification of HIPEC patients, i.e. personalised medicine, further research is warranted in this particular field. Another interesting finding is that both epithelial and stromal VCAN expression and a granular expression pattern are associated with a better resection outcome. An explanation for this, albeit speculative, might be related to the role of VCAN in creation of a microenvironment for tumour cells [21, 23, 24, 55–60, 64, 65]. In this way, VCAN plays a role in determining the consistency and appearance of tumour spots on the peritoneal surface, thereby possibly contributing to the extent to which these spots can be detected and subsequently resected.

In the current study, several possible markers were investigated in a study cohort consisting of HIPEC patients, representing a well-defined group of cancer patients with metastases confined to the peritoneum. In these patients, we found in the multivariate analysis high epithelial VCAN and VEGF expression to be associated with improved and worsened outcome respectively. To our knowledge, this is the first study examining stromal and epithelial VCAN expression in PM. We presume that higher VCAN expression is associated with improved sensitivity to chemotherapy, but this hypothesis should be further studied in vitro and in vivo and in well-defined patient cohorts. Further studies focusing of VCAN and other potential biomarkers could help improve selection of patients with PM, in this way minimising unnecessary exposure to the high morbidity and mortality rates associated with HIPEC treatment. A more comprehensive understanding of VCAN based mechanisms contributing to peritoneal dissemination and patient responses to therapy will potentially result in a more personalised approach in CRC patients with PM, ultimately leading to better treatment outcomes.

## Electronic supplementary material

Supplementary material 1 (DOCX 36 kb)

## Abbreviations

CRC: Colorectal carcinoma
PM: Peritoneal metastases
CRS: Cytoreductive surgery
HIPEC: Hyperthermic intraperitoneal chemotherapy
VCAN: Versican
VEGF: Vascular endothelial growth factor
MVD: Microvessel density
ECM: Extracellular matrix
MMC: Mitomycin C
PCI: Peritoneal cancer index
