# Supplementary material for: Versican and vascular endothelial growth factor expression levels in peritoneal metastases from colorectal cancer are associated with survival after cytoreductive surgery and hyperthermic intraperitoneal chemotherapy
Source: Clin Exp Metastasis. 2016 Feb 12;33:297–307. doi: 10.1007/s10585-016-9779-9 (PMC4799792; doi:10.1007/s10585-016-9779-9)
Supplement: Supplementary file 1 — Supplementary material 1 (DOCX 36 kb) [file 10585_2016_9779_MOESM1_ESM.docx]

**Supplementary table 1a** Associations between stromal versican (VCAN) expression, clinicopathological variables and other markers. ). CRS (cytoreductive surgery), HIPEC (hyperthermic intraperitoneal chemotherapy), PM (peritoneal metastases), simplified PCI (peritoneal cancer index), VEGF (vascular endothelial growth factor), MVD (microvessel density).

| **Stromal VCAN expression** | | **Low (%)^^** | **High (%)^^** | **p-value** |
| --- | --- | --- | --- | --- |
| Gender | Male | 10 (47.6) | 15 (34.1) | 0.414* |
|  | Female | 11 (52.4) | 29 (65.9) |  |
| Age at CRS and HIPEC (s.d.) | | 60.4 (13.4) | 59.3 (10.9) | 0.348^ |
| Tumour histology | Adenocarcinoma | 11 (52.4) | 28 (63.6) | 0.626* |
|  | Mucinous | 9 (42.9) | 13 (29.5) |  |
|  | Signet cell | 1 (4.8) | 3 (6.8) |  |
| Tumour differentiation | Well | 2 (16.7) | 1 (5.3) | 0.181* |
|  | Moderate | 6 (50.0) | 15 (78.9) |  |
|  | Poor | 4 (33.3) | 3 (15.8) |  |
| T-stage primary tumour | T1/T2 | 3 (15.0) | 0 (0) | 0.027* |
|  | T3/T4 | 17 (85.0) | 44 (100) |  |
| Stage primary tumour | Stage 1/2/3 | 6 (30.0) | 22 (50.0) | 0.178* |
|  | Stage 4 | 14 (70.0) | 22 (50.0) |  |
| Lymph node involvement | Negative | 2 (10.0) | 15 (34.9) | 0.065* |
|  | Positive | 18 (90.0) | 28 (65.1) |  |
| Location primary tumour | Colon | 18 (85.7) | 31 (70.5) | 0.525* |
|  | Rectum | 1 (4.8) | 4 (9.1) |  |
|  | Rectosigmoid | 2 (9.5) | 9 (20.5) |  |
| Synchronous PM | Yes | 15 (71.4) | 20 (45.5) | 0.065* |
|  | No | 6 (28.6) | 24 (54.5) |  |
| Simplified PCI | 1-4 | 15 (71.4) | 28 (63.6) | 0.587* |
|  | 5-7 | 6 (28.6) | 16 (36.4) |  |
| Resection outcome | R1 | 6 (28.6) | 31 (70.5) | 0.003* |
|  | R2 | 15 (71.4) | 13 (29.5) |  |
| **Associations with other markers** | | | | |
| Epithelial VCAN | Low | 11 (52.4) | 8 (18.2) | 0.008* |
|  | High | 10 (47.6) | 36 (81.8) |  |
| VCAN staining pattern | Negative | 5 (23.8) | 5 (11.4) | 0.471* |
|  | Not granular | 6 (28.6) | 14 (31.8) |  |
|  | Granular | 10 (47.6) | 25 (56.8) |  |
| VEGF | Low | 13 (61.9) | 23 (52.3) | 0.595* |
|  | High | 8 (38.1) | 21 (47.7) |  |
| MVD, % (s.d.) | | 22.4 (7.7) | 34.4 (12.6) | 0.001^ |

*Fisher’s exact test, ^Kruskal-Wallis test, ^^ Low: negative and weak expression, high: moderate and strong expression.

**Supplementary table 1b** Associations between epithelial versican (VCAN), clinicopathological variables and other markers. CRS (cytoreductive surgery), HIPEC (hyperthermic intraperitoneal chemotherapy), PM (peritoneal metastases), simplified PCI (peritoneal cancer index), VEGF (vascular endothelial growth factor), MVD (microvessel density).

| **Epithelial VCAN expression** | | **Low (%)^^** | **High (%)^^** | **p-value** |
| --- | --- | --- | --- | --- |
| Gender | Male | 6 (31.6) | 19 (41.3) | 0.579* |
|  | Female | 13 (68.4) | 27 (58.7) |  |
| Age at CRS and HIPEC (s.d.) | | 60.0 (12.5) | 59.5 (11.4) | 0.868^ |
| Tumour histology | Adenocarcinoma | 9 (47.4) | 30 (65.2) | 0.296* |
|  | Mucinous | 8 (42.1) | 14 (30.4) |  |
|  | Signet cell | 2 (10.5) | 2 (4.3) |  |
| Tumour differentiation | Well | 2 (25.0) | 1 (4.3) | 0.221* |
|  | Moderate | 4 (50.0) | 17 (73.9) |  |
|  | Poor | 2 (25.0) | 5 (21.7) |  |
| T-stage primary tumour | T1/T2 | 2 (11.1) | 1 (2.2) | 0.189* |
|  | T3/T4 | 16 (88.9) | 45 (97.8) |  |
| Stage primary tumour | Stage 1/2/3 | 7 (38.9) | 21 (45.7) | 0.781* |
|  | Stage 4 | 11 (61.1) | 25 (54.3) |  |
| Lymph node involvement | Negative | 2 (11.8) | 15 (32.6) | 0.121* |
|  | Positive | 15 (88.2) | 31 (67.4) |  |
| Location primary tumour | Colon | 15 (78.9) | 34 (73.9) | 0.639* |
|  | Rectum | 2 (10.5) | 3 (6.5) |  |
|  | Rectosigmoid | 2 (10.5) | 9 (19.6) |  |
| Synchronous PM | Yes | 11 (57.9) | 24 (52.2) | 0.787* |
|  | No | 8 (42.1) | 22 (47.8) |  |
| Simplified PCI | 1-4 | 12 (63.2) | 31 (67.4) | 0.779* |
|  | 5-7 | 7 (36.8) | 15 (32.6) |  |
| Resection outcome | R1 | 4 (21.2) | 33 (71.7) | <0.001* |
|  | R2 | 15 (78.9) | 13 (28.3) |  |
| **Associations with other markers** | | | | |
| Stromal VCAN | Low | 11 (57.9) | 10 (21.7) | 0.006* |
|  | High | 8 (42.1) | 36 (78.3) |  |
| VCAN staining pattern | Negative | 9 (47.4) | 1 (2.2) | <0.001* |
|  | Not granular | 10 (52.6) | 10 (21.7) |  |
|  | Granular | 0 | 35 (76.1) |  |
| VEGF | Low | 10 (52.6) | 26 (56.5) | 0.791* |
|  | High | 9 (47.4) | 20 (43.5) |  |
| MVD, % (s.d.) | | 22.9 (9.6) | 33.9 (12.4) | 0.007^ |

*Fisher’s exact test, ^Kruskal-Wallis test, ^^ Low: negative and weak expression, high: moderate and strong expression.

**Supplementary table 1c** Associations between vascular endothelial growth factor (VEGF), clinicopathological variables and other markers. CRS (cytoreductive surgery), HIPEC (hyperthermic intraperitoneal chemotherapy), PM (peritoneal metastases), simplified PCI (peritoneal cancer index), VCAN (versican), MVD (microvessel density).

| **VEGF expression** | | **Low (%)^^** | **High (%)^^** | **p-value** |
| --- | --- | --- | --- | --- |
| Gender | Male | 10 (27.8) | 15 (51.7) | 0.073* |
|  | Female | 26 (72.2) | 14 (48.3) |  |
| Age at CRS and HIPEC (s.d) | | 58.6 (11.8) | 60.8 (11.5) | 0.795^ |
| Tumour histology | Adenocarcinoma | 23 (63.9) | 16 (55.2) | 0.783* |
|  | Mucinous | 11 (30.6) | 11 (37.9) |  |
|  | Signet cell | 2 (5.6) | 2 (6.9) |  |
| Tumour differentiation | Well | 1 (6.7) | 2 (12.5) | 0.085* |
|  | Moderate | 13 (86.7) | 8 (50.0) |  |
|  | Poor | 1 (6.7) | 6 (37.5) |  |
| T-stage primary tumour | T1/T2 | 1 (2.8) | 2 (7.1) | 0.577* |
|  | T3/T4 | 35 (97.2) | 26 (92.9) |  |
| Stage primary tumour | Stage 1/2/3 | 7 (38.9) | 21 (45.7) | 0.781* |
|  | Stage 4 | 11 (61.1) | 25 (54.3) |  |
| Lymph node involvement | Negative | 10 (27.8) | 7 (25.9) | 1.000* |
|  | Positive | 26 (72.2) | 20 (74.1) |  |
| Location primary tumour | Colon | 26 (72.2) | 23 (79.3) | 0.908* |
|  | Rectum | 3 (8.3) | 2 (6.9) |  |
|  | Rectosigmoid | 7 (19.4) | 4 (13.8) |  |
| Synchronous PM | Yes | 16 (44.4) | 19 (65.5) | 0.133* |
|  | No | 20 (55.6) | 10 (34.5) |  |
| Simplified PCI | 1-4 | 23 (63.9) | 20 (69.0) | 0.794* |
|  | 5-7 | 13 (36.1) | 9 (31.0) |  |
| Resection outcome | R1 | 24 (66.7) | 13 (44.8) | 0.086* |
|  | R2 | 12 (33.3) | 16 (55.2) |  |
| **Associations with other markers** | | | | |
| Stromal VCAN | Low | 13 (36.1) | 8 (27.6) | 0.595* |
|  | High | 23 (63.9) | 21 (72.4) |  |
| Epithelial VCAN | Low | 10 (27.8) | 9 (31.0) | 0.791* |
|  | High | 26 (72.2) | 20 (69.0) |  |
| VCAN staining pattern | Negative | 5 (13.9) | 5 (17.2) | 0.888* |
|  | Not granular | 12 (33.3) | 8 (27.6) |  |
|  | Granular | 19 (52.8) | 16 (55.2) |  |
| MVD, % (s.d.) | | 32.0 (12.5) | 29.7 (12.8) | 0.596^ |

*Fisher’s exact test, ^Kruskal-Wallis test, ^^ Low: weak and moderate expression, high: strong expression.

**Supplementary table 1d** Associations between microvessel density (MVD), clinicopathological variables and other markers. CRS (cytoreductive surgery), HIPEC (hyperthermic intraperitoneal chemotherapy), PM (peritoneal metastases), simplified PCI (peritoneal cancer index), VCAN (versican), VEGF (vascular endothelial growth factor).

| **MVD** | | **Mean (s.d.)** | **p-value** |
| --- | --- | --- | --- |
| Gender | Male | 32.3 (11.5) | 0.517* |
|  | Female | 30.4 (13.3) |  |
| Age at CRS and HIPEC | | | 0.449^ (ρ=-0.102) |
| Tumour histology | Adenocarcinoma | 32.8 (13.6) | 0.358* |
|  | Mucinous | 27.9 (10.7) |  |
|  | Signet cell | 30.4 (11.7) |  |
| Tumour differentiation | Well | 24.5 (8.5) | 0.711* |
|  | Moderate | 29.1 (12.8) |  |
|  | Poor | 26.2 (12.6) |  |
| T-stage primary tumour | T1/T2 | 62.0 (15.0) | 0.122* |
|  | T3/T4 | 59.3 (11.5) |  |
| Stage primary tumour | Stage 1/2/3 | 58.0 (11.7) | 0.207* |
|  | Stage 4 | 60.5 (11.6) |  |
| Lymph node involvement | Negative | 59.3 (12.1) | 0.827* |
|  | Positive | 59.3 (11.6) |  |
| Location primary tumour | Colon | 31.4 (13.4) | 0.358* |
|  | Rectum | 36.0 (11.0) |  |
|  | Rectosigmoid | 27.2 (8.2) |  |
| Synchronous PM | Yes | 30.9 (10.9) | 0.749* |
|  | No | 31.2 (14.5) |  |
| PCI | 1-4 | 59.8 (11.9) | 0.757* |
|  | 5-7 | 59.3 (11.4) |  |
| Resection outcome | R1 | 33.2 (11.6) | 0.105* |
|  | R2 | 27.6 (13.6) |  |
| **Associations with other markers** | | | |
| Stromal VCAN | Low | 60.4 (7.7) | 0.001* |
|  | High | 59.3 (10.9) |  |
| Epithelial VCAN | Low | 22.9 (9,6) | 0.007* |
|  | High | 33.9 (12.4) |  |
| VCAN staining pattern | Negative | 24.3 (9.0) | 0.271* |
|  | Not granular | 33.6 (16.3) |  |
|  | Granular | 31.2 (10.6) |  |
| VEGF | Low | 32.0 (12.5) | 0.440* |
|  | High | 29.7 (12.8) |  |

*Kruskal-Wallis test, ^Spearman’s correlation coefficient
